# Preparation and Application of Egg Yolk Antibodies Against Chinese Sacbrood Virus Infection

**DOI:** 10.3389/fmicb.2018.01814

**Published:** 2018-08-03

**Authors:** Li Sun, Ming Li, Dongliang Fei, Qingyun Diao, Jian Wang, Liqin Li, Mingxiao Ma

**Affiliations:** ^1^Institute of Biological Sciences, Jinzhou Medical University, Jinzhou, China; ^2^Honeybee Research Institute, Chinese Academy of Agricultural Sciences, Beijing, China; ^3^Tianjin Speerise Challenge Biotechnology Co., Ltd., Tianjin, China

**Keywords:** Chinese sacbrood virus, egg yolk antibodies, IgY against CSBV, preparation of IgY, antibody titer, passive immunotherapy, controlling of CSBV infection

## Abstract

Chinese sacbrood virus (CSBV) infects *Apis cerana* larvae, resulting in the inability of the larvae to pupate and their consequent death, which may pose a serious threat to entire colonies. As there is no effective medical treatment for CSBV infections, further studies are necessary. In this study, an effective treatment for CSBV is described, based on a specific immunoglobulin Y (IgY) from egg yolk against CSBV. The inactivated vaccine was produced by ultracentrifugation and formalin treatment, using CSBV purified from a natural outbreak. The specific IgY was produced by immunization of white leghorn hens with the vaccine. An enzyme-linked immunosorbent assay using purified CSBV as the coating antigen revealed that the anti-CSBV IgY titer began increasing in the egg yolk on the 14th day post-immunization, reaching a peak on day 42, and anti-CSBV IgY remained at a high level until day 91. IgY isolated from the combinations of egg yolk collected between days 42–91 was purified by PEG and ammonium sulfate precipitation. In three repeated protection experiments using *A. cerana* larvae inoculated with CSBV, the survival rate of larvae was more than 80%, and the titer of anti-CSBV IgY was more than 2^5^ and 2^4^ when the larvae were fed IgY 24 h after and before inoculation with CSBV, respectively. Therefore, 400 colonies infected with CSBV were treated by feeding sugar containing IgY solutions with an antibody titer of 2^5^, and the cure rate was 95–100%. Three hundred susceptible colonies were protected by feeding the larvae with sugar containing IgY solutions with an antibody titer of 2^4^, and the protection rate was 97%. The results clearly suggest that a specific IgY was obtained from hens immunized with an inactivated-CSBV vaccine; this may be a novel method for controlling CSBV infection.

## Introduction

A large number of *Apis cerana* and *Apis* *mellifera* are raised in China, which provide a wealth of nutrient-rich beehive products and pollinate a wide variety of crops and flowers. However, honeybees often die or escape, and it is possible for the entire colony to collapse because of infection with pathogens such as viruses, bacteria, fungi, parasites, and protozoa (Schmid-Hempel and Schmid-Hempel, 1998; Liu et al., 2010). Among honeybee pathogens, Chinese sacbrood virus (CSBV) is the most serious threat to bee health and has caused widespread concern among beekeepers and researchers. CSBV primarily affects the brood of honeybees and results in larval death (Ghosh et al., 1999), as well as reducing the lifespan of adult bees (Bailey, 1969, 1976). Larvae that are between 1 and 3 days old are most susceptible to CSBV infection. CSBV infection causes death in infected larvae, before or after they are capped; the tissue of infected larvae becomes liquefied, their body surface changes color, and larvae dry out shortly after death (Yan and Han, 2008). CSBV infection occurs mainly in spring, when brood rearing begins. Currently, CSBV is widespread in China and Southeast Asia.

As CSBV causes the death of honeybees, and even the collapse of entire colonies, it is often referred to as “bee cancer” by beekeepers. Outbreaks of CSBV have reduced the number of *A. cerana* colonies in Liaoning Province, China, from 40,000 to less than 20,000 in 2008 (Ma et al., 2011). Since 2008, *A. cerana* have been frequently infected by CSBV in this region of China, greatly affecting the region’s apiculture (Zhang, 2012).

Currently, there are no specific treatments for CSBV infections of honeybees (Feng et al., 1998). Egg yolk antibodies known as immunoglobulin Y (IgY) have been widely used in the prevention and control of epidemic diseases, and for research purposes (Sotiropoulou et al., 2012; Vega et al., 2012; Han et al., 2014). Oral immunoglobulin therapy with egg yolk-derived antibodies against porcine transmissible gastroenteritis virus, porcine epidemic diarrhea virus (Huanzhong et al., 2012), white spot syndrome virus (Yong et al., 2006), and rotavirus (Sarker et al., 2007) immunoglobulin’s were previously shown to achieve significant therapeutic effects against viral infections in clinical trials. However, IgY has not previously been used to prevent and treat CSBV.

This study was conducted to develop an IgY against CSBV from egg yolk. The inactivated oil-adjuvant vaccine was prepared by isolating and identifying CSBV, after which laying hens were vaccinated three times. Hen eggs were collected, and the specific IgY was prepared from egg yolk. Yolk antibodies were used in clinical treatment and were found to have significant effects on CSBV.

## Materials and Methods

### Purification and Confirmation of CSBV

The infected *A. cerana* were collected from Kuandian, Liaoning Province (40° 43′ 46.80″ N, 124° 46′ 40.55″ E). For virus purification, after infected *A. cerana* were weighed, the larvae were placed in sterile water (1.5 times their volume), and completely homogenized using a mortar and pestle. Following the methodology described by Ming-xiao et al. (2010), CSBV was purified using cesium chloride gradient centrifugation. The supernatant was filtered first through a 0.45-μm cell filter, and then through a 0.22-μm cell filter. The purified CSBV was fed to 2- or 3-day-old *A. cerana* larvae for virus passage. Eight days after larvae were inoculated with CSBV, CSBV was isolated and purified again from infected larvae using the methods described above. Subsequently, CSBV was identified by reverse transcription (RT) polymerase chain reaction (PCR) methodology to exclude black queen cell virus (BQCV), acute bee paralysis virus (ABPV), chronic bee paralysis virus (CBPV), deformed wing virus (DWV), Kashmir bee virus (KBV), and Israeli acute paralysis virus (IAPV), following the method of Hu et al. (2016). The CSBV virus samples, after demonstrating that they did not contain other viruses in addition to CSBV, were stored at -80°C until further use.

### Preparation of the Inactivated Vaccine

The protein content of the concentrated virus was determined using a BCA Protein Assay Kit (Thermo Scientific, Waltham, MA, United States). The protein concentration of the viral concentrated solution was diluted to 0.3 mg/mL using 0.01 M phosphate-buffered saline (PBS), pH 7.4. After dilution, the viral solution was treated with 0.4% formalin for 24 h. Tween-80 was added at an 8% volume of the viral diluents, mixed with the viral diluents, and used as the water phase. The mixture of the 1:10 volume ratio between Span-80 and No.10 white-oil was used as the oil phase, after sterilization at 121.3°C. After homogenization by centrifugation at 8,000 × g for 10 min, the oil phase was slowly added to the water phase at a 3:1 volume ratio, and the mixture of the oil phase and water phase was homogenized by centrifugation at 10,000 × g for 5 min. The inactivated vaccine was produced as the W/O type emulsion.

### Immunization of Hens

In total, 150 white leghorn laying hens, 24 weeks of age, were immunized intramuscularly at three different sites with 1 mL of the inactivated vaccine; the immunization was performed three consecutive times, with 2-week intervals. Additionally, a negative control was established by using non-immunized hens. Blood and egg samples were collected from the time of first vaccination, and then once per week during the experimental period. The blood was allowed to clot at 24–25°C, and the centrifuged sera were stored at -20°C. The collected eggs were stored at 4°C before being used to prepare IgY. The antibody titer in the serum was monitored to confirm increased antibody levels after the booster doses. In total, 15 non-immunized hens were processed in a similar manner.

### Enzyme-Linked Immunosorbent Assay

An enzyme-linked immunosorbent assay (ELISA) was conducted to check the serum antibody titer. The 96-well microtiter plates (Gibco, Grand Island, NY, United States) were coated overnight at 4°C with PBS (pH 7.4), containing 1 μg/mL of the purified CSBV antigen. CSBV-coated plates were washed three times with 0.05% Tween-20 in PBS (PBST). The wells were blocked with 300 μL of PBS containing 3% skim milk for 1 h at 37°C. The wells were washed again three times with PBST. Next, 100 μL of appropriately diluted serum (1:1000 dilution) preparations from immunized and non-immunized hens at different time intervals were added to the wells. The plates were then incubated at 37°C for 1 h, and washed three times with PBST, before adding horseradish peroxidase-conjugated rabbit anti-chicken IgG (Sigma Chemical Co., St. Louis, MO, United States) diluted (1:5,000) in PBS, and incubating for 45 min at 37°C. 3,3′,5,5′-Tetramethylbenzidine substrate solution was added, and plates were incubated for 15 min, before sulfuric acid was added to stop the reaction. The optical density (OD) was measured on an ELISA plate reader, using a 450-nm filter, and the ODs were statistically analyzed using statistical software SPSS17.

### Purification of IgY Antibodies From Egg Yolk

When the titer of the serum antibody was relatively stable, IgY antibodies were purified from combinations of the egg yolk collected. IgY antibodies were extracted from the yolk, which was separated from the white using an egg separator once a week from days 42 to 91 after the first immunization, according to the methods described by Polson et al. (1980) and Ko and Ahn (2007). Briefly, a volume of yolk was added to a 1/3 volume of buffer containing 14% polyethylene glycol (PEG) 6000 (w/v), and was stirred at 22–26°C for 30 min. After the mixture was centrifuged at 5,000 ×*g* for 20 min at 10°C, the supernatant (filtered through four layers of sterile gauze) was collected. Subsequently, PEG6000 was added to the filtrate with gentle stirring to adjust the final polymer concentration to 12% (w/v). The pellet was collected by centrifugation, and dissolved in the original volume of yolk in PBS to prepare the solution containing IgY. Solid ammonium sulfate was dissolved in the solution containing IgY, adjusted to 50% saturation, and stirred for approximately 12 h at 4°C. Collected centrifuged pellets were washed with 33% saturated ammonium sulfate. The content was desalted by dialysis. Purified IgY antibodies were sterilized with a 0.22-μm membrane filter and stored at -20°C.

Purified IgY antibodies were diluted by 2^2^, 2^3^, 2^4^, 2^5^, 2^6^, 2^7^, 2^8^, and 2^9^, and the titer of combinations of IgY antibodies against CSBV were determined by the above described ELISA method once a week from days 42 to 91. The IgY antibodies titer were statistically analyzed by the OD of same dilutions at different time intervals as a group, using the statistical software SPSS 17.

### SDS-PAGE

As described previously (Sentila et al., 2013), the above purified IgY antibodies were determined by sodium dodecyl sulfate polyacrylamide gel electrophoresis (SDS-PAGE) (Hatta et al., 1997), with a 4% stacking gel and 10% separating gel.

### Viral Neutralization Assay

The VN assay, used to detect the ability of IgY antibodies to neutralize CSBV, was modified from the mammalian VN assay protocol based on previously described methods (OIE, 2010.). The CSBV VN assay was performed as follows: First, CSBV were incubated with two-fold serial dilutions of IgY antibodies for 1 h at 37°C. Next, a total of 140 2-day-old larvae were retrieved from the same colony in Jinzhou Medical University Farm (41.12°N, 121.15°E), following Hu et al. (2016), and randomly distributed into seven groups, with each group containing 20 larvae, and then each larva in groups 1–6 was fed 10 μL of virus and IgY suspension containing 1.25 × 10^7^ copies of CSBV to reach the 100% mortality rate (Hu et al., 2016) and the IgY titer at 2^2^, 2^3^, 2^4^, 2^5^, and 2^6^, mixed with an equal amount of basic larval diet (BLD; 37% sterile water, 6% fructose, 50% royal jelly, 6% glucose, and 1% yeast extract) (Liu and Zeng, 2010), which was subsequently used for daily feeding. Group 7 served as a control. Larvae were kept under 95% relative humidity at 34°C. The virus neutralization titers were read as the last serum dilution showing protection of the larva. The above assay was performed in triplicate. The dead larvae were tested with RT-PCR for the following viruses during the VN assay: BQCV, ABPV, CBPV, DWV, KBV, IAPV, and CSBV.

### Protection of *A. cerana* Larvae Inoculated With CSBV

Two-days-old *A. cerana* larvae were used to verify the protective effect of IgY against CSBV. A total of 280 2-day-old larvae were retrieved from the same colony, following the method of Hu et al. (2016), and randomly distributed into 14 groups (termed groups 8–21), with each group containing 20 larvae. Groups 8–13 were inoculated with CSBV at 1.25 × 10^7^ copies/larva (Hu et al., 2016). Group 14 and 21 served as virus-free controls. Groups 15–20 were fed with anti-CSBV-specific IgY titer at 2^2^, 2^3^, 2^4^, 2^5^, and 2^6^ (after two-fold serial dilutions), and no anti-CSBV-specific IgY, respectively. To evaluate the curative effect on CSBV using the specific IgY, groups 8–13 were fed with anti-CSBV-specific IgY titer at 2^2^, 2^3^, 2^4^, 2^5^, and 2^6^ (after two-fold serial dilutions), and no anti-CSBV specific IgY after inoculating with CSBV 24 h, respectively. To evaluate the preventative effect on CSBV using the specific IgY, groups 15–20 were inoculated with CSBV at 1.25 × 10^7^ copies/larva, 24 h after being inoculated with IgY. The above assay was performed in triplicate.

Each larva was fed 10 μL of virus or IgY suspension mixed with an equal BLD, and was reared according to the above described rearing protocol.

The dead larvae were detected by RT-PCR for the following viruses during the protection experiments: BQCV, ABPV, CBPV, DWV, KBV, IAPV, and CSBV.

### Application of Egg Yolk Antibodies

Four hundred colonies infected with CSBV were diagnosed by clinical symptoms or RT-PCR detection from May to August in 2013 and 2014 in Huairen, Liaoning Province (40.76°N, 120.85°E); Kuandian, Liaoning Province (40.75°N, 124.77°E); and Chengde, Hebei Province (40.95°N, 117.96°E). Of the 400 CSBV-infected colonies, 20 colonies escaped because of the loss of the queen and drone spawn, and 153 colonies were confirmed to be CSBV-positive by RT-PCR. If, in a Colony, (1) capped brood was not normally sealed, became dark black, down sunken and perforated, (2) there are a large number of sick and dead larvae in the honeycomb with the capped brood not normally sealed, (3) around the entrance of the colonies, even very few capped brood sealed and larvae were observed, and (4) the production capacity of worker bees to gather honey and pollen were reduced and disordered, it was designated as serious infection groups. If, in a colony, (1) CSBV infection was detected by RT-PCR analysis, (2) the larvae infected showed head warped upward, body surface change white, but (3) the production capacity of worker bees to gather honey and pollen was normal and orderly, very few capped broods were not normally sealed, and (4) no sick and dead larvae were observed around the entrance of the colonies, it was designated the moderate infection group (*n* = 227 colonies). The reasons why the dead larvae were not observed were probably that there were a smaller number of dead larvae, and they were cleaned by worker bees before observation. Among the 400 CSBV-infected colonies, there were four escaped colonies, 39 serious infection colonies, and 60 moderate infection colonies in Huairen, Liaoning Province; 11 escaped colonies, 71 serious infection colonies, and 115 moderate infection colonies in Kuandian, Liaoning Province; and five escaped colonies, 43 serious infection colonies, and 52 moderate infection colonies in Chengde, Hebei Province. The aforementioned 400 CSBV-infected colonies were treated using purified IgY diluted to a titer of 2^5^ with PBS containing sugar. The solution was fed to infected *A. cerana* (20 mL day^-1^ colony^-1^) by direct feeding with continuous feeding three times, followed by feeding once a day. Five *A. cerana* larvae (4 to 6-days-old) were randomly selected from serious infection groups and moderate infection group on 2, 3, 4, 5, 6, 7, and 8 days after treatment using purified IgY in Huairen, Liaoning Province, Kuandian, Liaoning Province, and Chengde, Hebei Province, respectively; totally 210 *A. cerana* larvae were collected and subjected to RT-PCR analysis for CSBV.

Three hundred susceptible colonies that had previously been infected with CSBV, but 1 year later, in which CSBV was not detected by RT-PCR, were protected using purified IgY before the brood rearing began in March 2015 in Kuandian, Liaoning Province. IgY was diluted to a titer of 2^4^ with PBS, and then sugar was dissolved in the solution. The solution was fed to 300 healthy *A. cerana* colonies (20 mL day^-1^ colony^-1^) by direct feeding with continuous feeding three times, followed by daily feeding.

Honeybee belongs to the social insect, which there are about 20–40 thousands of honeybee in a colony, a queen spawns more than 1,000 eggs a day, and the normal lifetime of honeybee is about a month or so during the breeding period. So the number of honeybee cannot be accurately counted. Therefore, the standard for the recovery of colonies infected with CSBV were as follows:

(a) No sick and dead larvae were observed in a honeycomb and around the entrance of the colonies.
(b) The production capacity of worker bees to gather honey and pollen was normal and orderly.
(c) More than 50% capped brood were normally sealed and connected to one another as a whole.
(d) CSBV could not be detected using RT-PCR.

### Stability of IgY in Terms of Route of Oral

In total, 20 worker honeybees were sacrificed for tissue dissection, respectively. Each worker honeybee was fixed onto the wax top of a dissecting dish with insect pins, and the abdomen was opened with scissors. Honey sac were carefully separated and removed with forceps under a dissecting microscope. The contents of the honey sac are sucked out with syringe (volume 1 mL), and dripped into 200 μL of appropriately diluted IgY titer at 2^5^ (equal to the dose used to treat 20 bees), respectively. The IgY interacted with the contents of the honey sac for 12 and 24 h at 37°C. Then, the supernatants of these suspensions were separated by centrifugation at 8,000 ×*g*, and subjected to subsequent IgY titer detection using ELISA. Additionally, the same operation was performed to detect the IgY titer for BLD containing IgY titer at 2^5^ and IgY antibody original solution titer at 2^5^.

## Results

### Determination of Serum Antibody Titers

**FIGURE 1:**
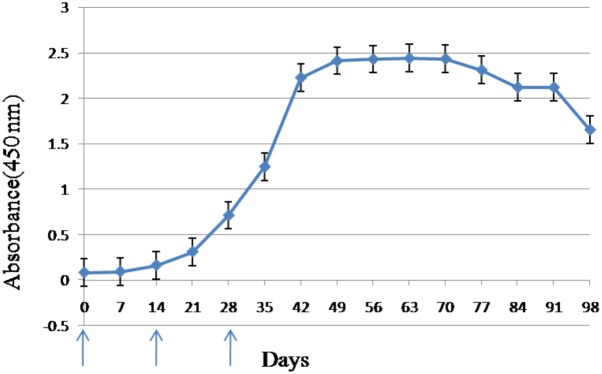
Antibody response in serum of laying hens immunized with CSBV. Anti-CSBV activity was determined by ELISA. The specific antibody levels began increasing in the serum 14 days after the first immunization, reaching a peak on day 42, and remaining high until day 91. *Arrows* represent the time of immunization; *bars* represent SE.

The ELISA assay revealed that the anti-CSBV antibody titer began to rise in the sera of inoculated white leghorn hens 14 days after the first immunization. The anti-CSBV antibody titer peaked on day 42 and was then maintained at high levels from days 42 to 91 (**Figure 1**). Eggs were collected from days 42 to 91.

### Titer of Combinations of IgY Antibodies Against CSBV by ELISA

**FIGURE 2:**
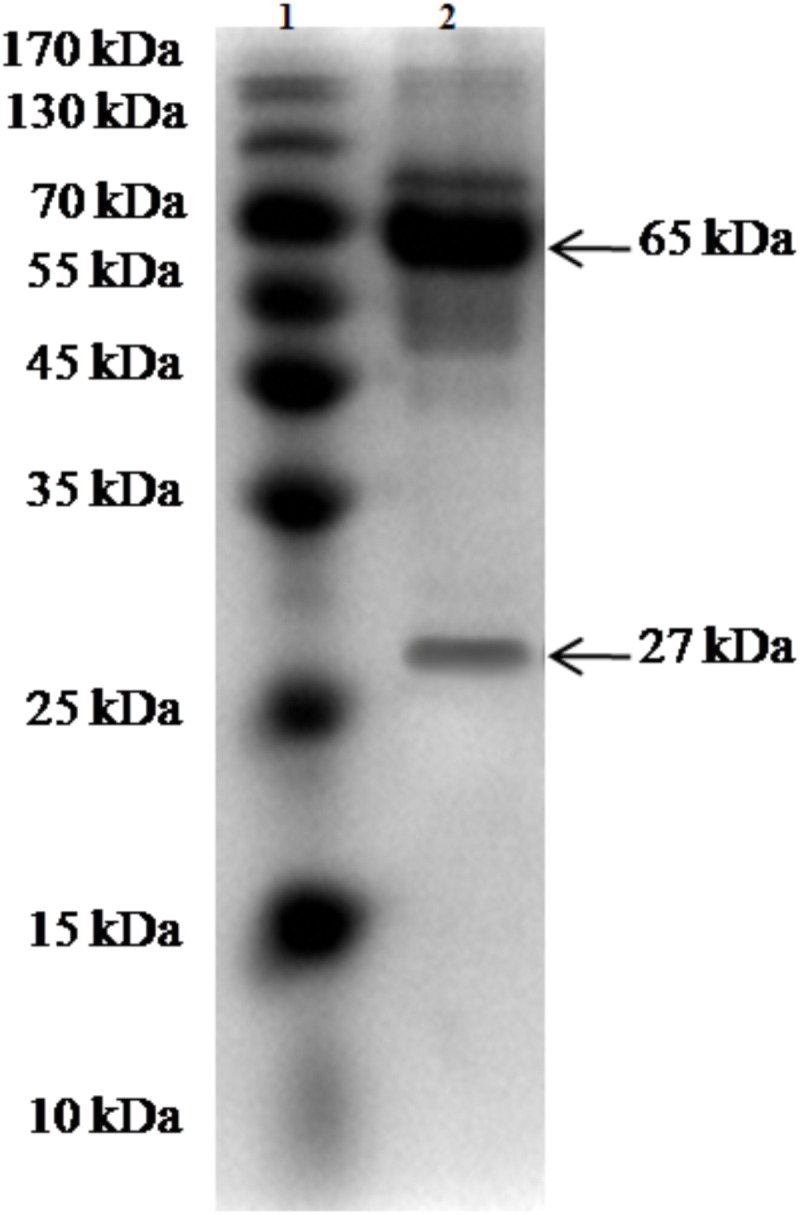
SDS-PAGE of the purified IgY for CSBV. *Lane 1*: Protein marker and *Lane 2*: Purity of IgY.

**FIGURE 3:**
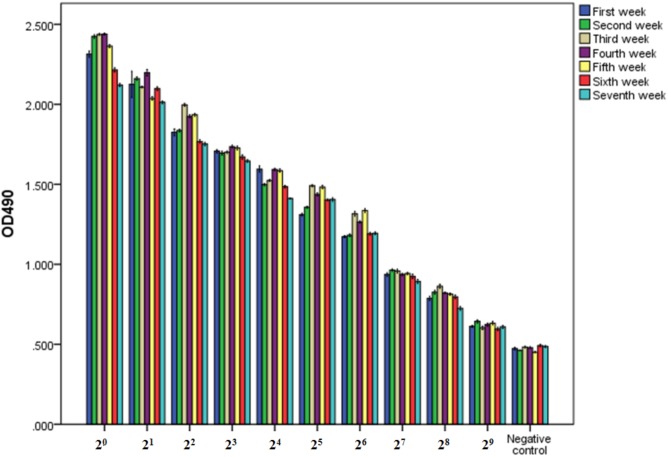
Titer of combinations of IgY antibodies against CSBV detected by ELISA once a week from days 42 to 91 after the first immunization. The OD ratio of combinations of anti-CSBV specific IgY to the negative control was >1.5 at 22, 23, 24, 25, 26, 27, and 28 dilutions, while the 29 dilution was <1.5. The results indicated that the titer of combinations of anti-CSBV-specific IgY is 28. *Bars* represent SE.

The purity and molecular weights of IgY antibodies against CSBV were determined by SDS-PAGE. Under reduced conditions, the IgY antibodies against CSBV presented two bands of approximately 65 and 27 kDa, respectively (**Figure 2**). Using ELISA, the OD ratio of combinations of anti-CSBV specific IgY to the negative control was >1.5 at 2^2^, 2^3^, 2^4^, 2^5^, 2^6^, 2^7^, and 2^8^ dilutions, while the 2^9^ dilution was <1.5. The results showed that the titer of combinations of anti-CSBV specific IgY was 2^8^ (**Figure 3**).

### VN Assay

Triplicate experiments revealed that the mortality rates of the larvae were 65–75% (14/20, 12/20, and 15/20), 40–50% (10/20, 9/20, and 8/20), 25–35% (7/20, 5/20, and 7/20), 10–20% (3/20, 2/20, and 4/20), 10–20% (2/20, 4/20, and 2/20), 100% (20/20), and 10–20% (3/20, 3/20, and 4/20) in groups 1–7, respectively, and these larvae died before pupation (**Table 1**), but the death time of larvae in groups 2 and 3 tended to be delayed.

**Table 1:** Viral neutralization assay.

Group	The number of dead larvae at different time points(a/b/c)				
	24 h	48 h	72 h	96 h	120 h
1	3/1/0	2/4/4	7/3/7	2/4/4	0/0/0
2	2/0/1	0/3/1	3/3/2	5/2/3	0/1/1
3	0/1/0	1/0/3	0/0/1	3/2/0	3/2/3
4	1/0/1	0/1/0	1/2/1	0/1/0	0/0/0
5	0/1/1	1/1/0	2/0/0	0/2/1	0/0/0
6	1/2/1	8/9/12	9/8/7	2/1/0	0/0/0
7	2/0/1	1/1/1	0/2/1	0/1/0	0/0/0


*a, b, and c represent the number of dead larvae from three repeated experiments.*

All dead larvae found during the VN assay were analyzed by RT-PCR, and the results showed that all of the other honeybee viruses were undetectable, whereas CSBV was detectable.

### Protection of *A. cerana* Larvae Inoculated With CSBV

Triplicate experiments revealed that the mortality rates of the larvae were 85–95% (19/20, 19/20, and 17/20), 60–70% (12/20, 14/20, and 12/20), 35–50% (8/20, 10/20, and 7/20), 20–30% (5/20, 4/20, and 6/20), 10–15% (2/20, 3/20, and 3/20), 100% (20/20), and 10–15% (2/20, 3/20, and 2/20) in groups 8–14, respectively, and these larvae died before pupation (**Table 2**), but the death time of larvae in groups 10 and 11 tended to be delayed. The survival rate was not 100% because of the death of larvae due to artificial circumstances.

**Table 2:** The curative effect on CSBV of using the specific IgY.

Group	The number of dead larvae at different time points(a/b/c)				
	24 h	48 h	72 h	96 h	120 h
8	5/4/3	9/8/5	4/7/7	1/0/2	0/0/0
9	3/2/3	3/4/4	5/6/3	1/2/2	0/0/0
10	1/0/2	2/1/0	3/4/1	1/5/4	1/0/0
11	2/1/1	1/0/1	1/2/2	1/0/0	0/1/2
12	0/0/0	1/2/2	0/1/0	1/0/1	0/0/0
13	8/9/9	7/8/6	4/3/3	1/0/2	0/0/0
14	2/1/0	0/2/1	0/0/0	0/0/1	0/0/0


*a, b, and c represent the number of dead larvae from three repeated experiments.*

Three repeated experiments indicated that the mortality rates of the larvae were 60–80% (15/20, 12/20, and 16/20), 45–55% (11/20, 11/20, and 9/20), 25–40% (7/20, 5/20, and 8/20), 10–20% (2/20, 4/20, and 2/20), 10–20% (3/20, 4/20, and 2/20), 100% (20/20), and 10–20% (2/20, 4/20, and 3/20) in groups 15–21, respectively, and these larvae died before pupation (**Table 3**) but the death time of larvae in group 17 tended to be delayed.

**Table 3:** The preventative effect on CSBV of using the specific IgY.

Group	The number of dead larvae at different time points(a/b/c)				
	24 h	48 h	72 h	96 h	120 h
15	4/1/0	2/4/3	6/3/8	3/4/5	0/0/0
16	2/0/1	1/2/2	3/5/3	5/4/3	0/0/0
17	0/1/0	1/0/3	0/1/1	3/2/1	3/1/3
18	2/0/0	0/1/1	0/2/1	0/1/0	0/0/0
19	0/1/1	1/1/0	2/0/0	0/2/1	0/0/0
20	1/2/1	8/9/12	9/8/7	2/1/0	0/0/0
21	2/0/1	1/1/0	0/2/1	1/0/0	0/0/0


*a, b, and c represent the number of dead larvae from three repeated experiments.*

All dead larvae found during the protection experiments were analyzed by RT-PCR, and the results showed that all of the other honeybee viruses were undetectable, whereas CSBV was detectable.

### Application of Egg Yolk Antibodies

**FIGURE 4:**
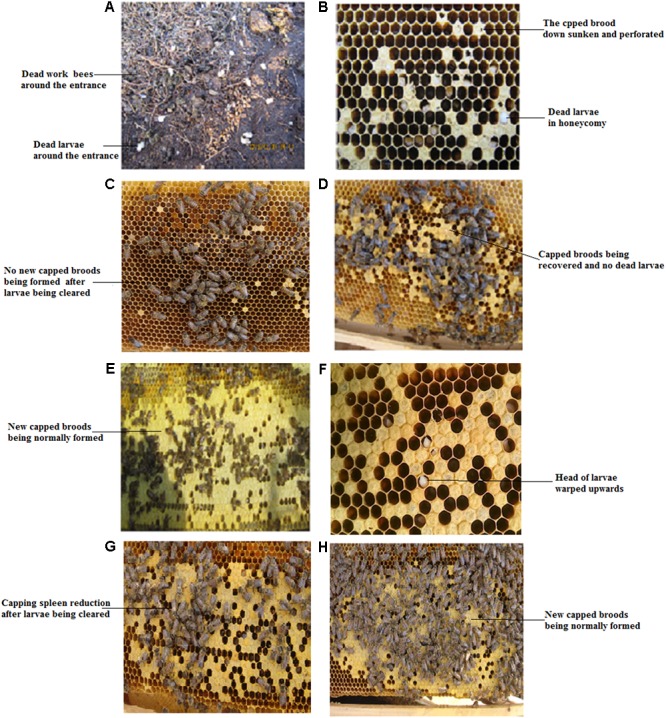
Clinical symptoms of *Apis cerana* larvae infected with CSBV, and effect of treatment with anti-CSBV IgY. **(A)** Numerous larvae were dragged out of the colony, and dead worker bees were observed in the serious infection group. **(B)** Capped broods not being normally sealed, down sunken and perforated, were observed in the serious infection group. **(C)** No new capped broods being formed after larvae infected with CSBV being cleared in the serious infection group. **(D)** New capped broods begin to be formed, and no larvae were dragged out of the colony from 7 to 13 days after IgY treatment in the serious infection group. **(E)** The heads of larvae infected with CSBV were warped upward. **(F)** The capped brood was normally sealed from 18 to 21 days after IgY treatment. **(G)** Capping spleen reduction after larvae infected with CSBV being cleared in the moderate infection group. **(H)** New capped broods were normally formed 10 to 14 days after IgY treatment in the moderate infection group.

In the 400 CSBV-infected colonies, 20 colonies were not treated because of loss of the queen and drone spawn. In the 165 colonies in the serious infection group, no sick and dead larvae were observed in a honeycomb and around the entrance of the colonies (Conforming to standard a), and the production capacity of worker bees to gather honey and pollen restored from days 7 to 13 after treatment with IgY (Conforming to standard b), and the capped broods were normally formed and connected to one another as a whole(Conforming to standard c) from days 18 to 21 after treatment with IgY (**Figures 4A–D**). CSBV could be detected using RT-PCR from days 2 to 6, but could not be detected from the 7th day (**Table 4**), (Conforming to standard d), and the capped broods were normally formed after treatment purified IgY in Huairen, Liaoning Province and Chengde, Hebei Province, respectively. CSBV could be detected using RT-PCR from days 2 to 5, but could not be detected from the 6th day (**Table 4**), and the capped broods were normally formed, after treatment with purified IgY in Kuandian, Liaoning Province. In the 215 colonies in the moderate infection group, no larvae whose head warped upward and body surface change white were observed (Conforming to standard a), new capped broods were normally formed 10 to 14 days after treatment with IgY (**Figures 4E–H**). CSBV could be detected using RT-PCR from days 2 to 3, but could not be detected from the 4th day (**Table 4**) (Conforming to standard d), and the capped broods were normally formed after treatment with purified IgY in the three bee farms (Conforming to standard b). The infected swarm bees were cured after approximately 1 month, and the cure rate was 95–100% according to the standard for the recovery of colonies infected with CSBV.

**Table 4:** A total of 210 *A. cerana* larvae were randomly selected to detect CSBV by RT-PCR.

Geographic location	Classification of colony infected	Number of samples	Ratio of positive to negative samples at different time points from 2 to 8 days (a/b)						
			2	3	4	5	6	7	8
Huairen	Serious infection	35	5/0	5/0	5/0	5/0	2/3	0/5	0/5
	Moderate infection	35	5/0	1/4	0/5	0/5	0/5	0/5	0/5
Kuandian	Serious infection	35	5/0	5/0	2/3	1/4	0/5	0/5	0/5
	Moderate infection	35	5/0	2/3	0/5	0/5	0/5	0/5	0/5
Chengde	Serious infection	35	5/0	5/0	5/0	3/2	1/4	0/5	0/5
	Moderate infection	35	5/0	1/4	0/5	0/5	0/5	0/5	0/5


*a and b represent the number of positive and negative samples for detecting CSBV by RT-PCR, respectively. Number of sample 35 means that 5 *A. cerana* larvae were randomly selected on 2, 3, 4, 5, 6, 7, and 8 days after treatment.*

From March to September 2015, which is the period of *A. cerana* breeding in Kuandian, Liaoning Province, 291 of 300 colonies subjected to prevention treatment with purified IgY bred normally, produced honey, and pollinated plants, while the other nine colonies were diagnosed with CSBV infections, as detected by RT-PCR. The capped broods were not normally sealed, and no larvae were removed from the colony. These results indicate a protection rate of 97%.

### Stability of IgY in Terms of Route of Oral

Comparison with IgY antibody original solution, the titer of IgY antibody interacting with the contents of the honey sac and BLD for 12 and 24 h showed no differences; they were still 2^5^.

## Discussion

In China, CSBV frequently infects *A. cerana*, and negatively affects the region’s apiculture. This virus can also cross the species barrier to cause the death of *A. mellifera* larvae (Sun et al., 2017). Additionally, SBV infections of *A. mellifera* are widespread and cause disease worldwide, influencing the production of bee products and plant pollination (Cavigli et al., 2016; Desai and Currie, 2016; Tsevegmid et al., 2016).

In recent years, some CSBV prevention and control schemes have been studied, including feeding *A. mellifera* with royal jelly, silver ion, CSBV dsRNA, and SP6 (Zhang et al., 2014, 2016; Ahn et al., 2015; Shen et al., 2017). However, these measures are still in the research stage, and CSBV infections have not been completely controlled. Traditional Chinese medicine treatment may be useful for the control of CSBV, but the effects of such treatment are slow, and traditional Chinese medicines have a poor cure rate for serious infection colonies, likely because these measures do not directly inactivate the virus, but rather improve resistance to the virus through immune regulation (Hanrong, 2000). Furthermore, *A. cerana* often refuse to feed on traditional Chinese medicines or avoid them due to the strong odor, which may result in the entire colony being destroyed. Specific IgY antibodies are increasingly used in passive immunotherapy for infectious diseases in recent years, and IgY antibodies are safe, inexpensive, and have no known side effects.

Mainly, five factors influence the development and production of specific IgY: The dose and molecular weight of the target antigen, the type of adjuvant used, the route of application, the immunization frequency, and the interval between immunizations (Schade and Hlinak, 1996). In this study, the titer of IgY antibodies against CSBV began increasing in the serum on day 14 after the first immunization, reaching a peak on day 42, and then remained at a high level until day 91, and the titer of combinations of anti-CSBV specific IgY was 2^8^ from days 42 to 91. These results suggest that the collected eggs can be used to produce a large amount of IgY from days 42 to 91 after the first immunization, and the target antigen (inactivated CSBV vaccine) and immunization program, using continuous immunization at 2-week intervals, was suitable for preparing anti-CSBV IgY.

Various IgY extraction methods were reviewed in detail by De Meulenaer and Huyghebaert (2001); for review, see Schade et al., 2005). These methods include the water dilution methods (Kim and Nakai, 1996), ammonium or sodium sulfate precipitation method (Akita and Nakai, 1992), PEG method precipitation method (Polson et al., 1985; Polson, 1990), dextran sulfate precipitation method (Jensenius et al., 1981), precooled propane and acetone method (Bade and Stegemann, 1984), and water dilution ultrafiltration method (Chen, 2010). In order to select a suitable IgY extraction method, important influencing factors should be taken into account, including the scale of extraction (laboratory or industrial), cost effectiveness, production equipment, and impact on the environment (waste management). Akita and Nakai (1993) compared the water dilution methods to other methods in terms of yield purity and activity of IgY. The water extraction of IgY is sufficient to achieve good results, but Pauly et al. (2011) reported that IgY-extraction by means of PEG-precipitation is very cost-effective and results in highly specific antibodies with a stable titer, and worked very well in a large number of different immunological assays.

The classical VN assay must be performed on susceptible animals (including chicken embryos) or cells, but CSBV cannot be cultivated *in vitro* because of the lack of appropriate culture cells. Additionally, only larvae are used in CSBV cultures, and they may die naturally under the conditions of artificial rearing. The protocol of the CSBV neutralization assay have to be modified based on previously described methods (OIE, 2010). In the three repeated VN assays, the mortality rate of the larvae was less than 20% when the titer of the anti-CSBV IgY was more than 2^4^. A 0% mortality rate was not achieved because the artificial rearing conditions were not identical to the natural growth conditions of honey bees, and because there are individual differences between larvae. This may also explain why larval mortality was not exactly the same in the three replicated experiments. Nonetheless, these results indicate that the survival rate of larvae that were fed with a mixture of CSBV and IgY interacting with each other (IgY at a certain titer and neutralized with CSBV) was consistent with that of larvae not inoculated with the virus. Thus, CSBV can completely neutralized by anti-CSBV IgY at a certain titer, and when less than the optimum titer, CSBV was partially neutralized causing to the survival time of the larvae to be prolonged. Similar experimental results were revealed in the three repeated protection experiments of *A. cerana* larvae inoculated with CSBV. These results showed that anti-CSBV IgY can protect *A. cerana* larvae exposed to CSBV. Following this result, IgY was used to treat 400 infected bee colonies, and to prevent infection in 300 susceptible bee colonies. The cure rate was more than 95%, and the protection rate was more than 97%. The clinical applications show that IgY treatment of CSBV is fast-acting, effective, and specific, and has a high cure rate, with rapid recovery. Furthermore, IgY directly and rapidly binds to the virus; thus, IgY can be used for the emergency prevention of CSBV outbreaks.

Orally administered antibodies, like any other protein, are susceptible to denaturation by the acidic pH of the stomach and degradation by proteases (Hatta et al., 1993; Shimizu et al., 1993) in mammals and poultry. But IgY is relatively tolerant to pressure (4,000 kg/cm^2^), high temperature (75–80°C), and low pH value (pH = 3) as reported, with no detectable inactivation of IgY in aqueous solutions with high sugar concentrations (Shimizu et al., 1994). In addition to increasing the fraction of immune reactive antibodies delivered locally into the gastrointestinal tract, microencapsulation has been used to protect IgY from gastrointestinal inactivation (Chang et al., 2002; Cho et al., 2005; Kovacs-Nolan and Mine, 2005) in mammals and poultry. In the present study, we observed that the IgY antibodies titer did not change in an *in vitro* simulation experiment of honey bees, and the specific anti-CSBV IgY antibodies could effectively prevent and control CSBV by orally administered antibodies. The reason for this may be that the physiological conditions of the digestive system and characteristics of digestibility and absorption of honey bee are different from those of mammals and poultry, or high sugar concentrations of the honey sac and BLD enhanced the stability of the IgY (Shimizu et al., 1994), but the mechanism of CSBV being prevented and treated by IgY should be studied in the future.

In summary, the results of this study indicated that “universal” passive immunotherapy using a specific IgY for CSBV is a novel method for controlling CSBV infections. These results also indicated that a large amount of low-cost specific antibodies can be produced using egg yolks from immunized laying hens. We will continue researching the mechanism underlying the action of IgY against CSBV in our laboratory.

## Ethics Statement

The use of the experimental animals involved in the article is in compliance with the relevant provisions of the Animal Welfare and Ethics of Experimental Animals of the Experimental Animal Center of Jinzhou Medical University, China.

## Author Contributions

LS and MM designed the study and wrote the manuscript. LS, ML, DF, QD, JW, LL, and MM performed the experiments and analyzed the data. All authors approved the final version of the manuscript.

## Conflict of Interest Statement

The authors declare that the research was conducted in the absence of any commercial or financial relationships that could be construed as a potential conflict of interest.
